# Antioxidant, antimicrobial, and apoptosis-related activities of *Azadirachta indica* (Neem) leaf extract in MCF-7 and A549 cell lines

**DOI:** 10.1038/s41598-026-48147-5

**Published:** 2026-04-26

**Authors:** Elshahat A. Toson, Mohammed Marzouk, Norhan A. Rezk, Sami Rabei, Rasha F. Zahran

**Affiliations:** 1 Chemistry Department, Faculty of Science, Damietta University, New Damietta, 34517 Egypt; 2 Botany and Microbiology Department, Faculty of Science, Damietta University, New Damietta, 34517 Egypt; 3 Chemistry Department, Faculty of Science, Damietta University, New-Damietta, Damietta 34517 Egypt

**Keywords:** Neem, Antioxidant, Antimicrobial, Cytotoxicity apoptosis, Biochemistry, Biotechnology, Drug discovery, Microbiology, Plant sciences

## Abstract

This study aimed to extract neem leaves components to determine their antimicrobial and antioxidants activities. In addition, their ability to suppress the proliferation of A549 and MCF-7 cells were studied to explore the possible underlying mechanisms involved during such effects. Therefore, the antimicrobial activity of the extract towards some bacterial and fungal strains was investigated using agar well diffusion method, minimum inhibition concentration (MIC) test, peroxidase (POX), and catalase (CAT) activities. Superoxide dismutase (SOD) and catalase (CAT) like activities as well as total antioxidant capacity (TAC) and DPPH scavenging activity were determined. Cells viabilities were also measured by the mitochondrial-dependent reduction of MTT on A549 and MCF-7 cell lines. Cell cycle was flowcytomerically analyzed to detect which phase was arrested. Quantitative real-time PCR (RT-PCR) was done to assess the changes in apoptotic and anti-apoptotic genes. Results showed that, neem leaves extract was more effective towards Gram-negative bacteria (*E. aerogenes* and *E. coli*) than that of Gram-positive type (*B. cereus* and *S. epidermidis*). Also, the extract has a high antioxidant activity compared to that of the ascorbic acid (DPPH IC_50_ was 0.22 and 0.51 mg/ml for extract and ascorbic acid, respectively). Cytotoxicity assays confirm that the extract is able to suppress the proliferation of cell lines in a dose-dependent manner with IC_50_ of 128.1 and 55.7 µg/ml for A549 and MCF-7, respectively. The extract promotes arrest of cell cycle at G0, inhibited the anti-apoptotic genes and stimulated the pro-apoptotic ones. In conclusion, neem leaves extract could have promising roles in pharmaceutical and medical applications.

## Introduction

*Azadirachta indica* (Neem) present in many tropical countries^1^. Its leaves extracts contain different phytochemicals including alkaloids, triterpenoids, glycosides, tannins, saponins, and steroids^2^. These may be responsible for their medicinal and physiological actions on the human body. Neem leaf extract and their phytochemical constituents have demonstrated activity against infectious diseases and metabolic disorders, including diabetes, and may also possess anticancer potential^3^. This revision enhances precision and readability.They also possess immune-modulatory, antimicrobial, anti-inflammatory properties and antioxidant activity^4,5^.

The toxicity of neem components towards the normal cells have always been an issue in its therapeutic application. In previous studies, it was proved that neem extract had a cytotoxic effect on different cancer cell lines but it showed no significant toxicity against normal cells in-vitro^6^.

The flavonoids present in neem plant may represent its ability to play a role in their activity against different cancer cells^7^. The phenolic contents of neem; which include catechins and other flavonoids, may help to mediate its antitumor properties^8^. These properties are related with many molecular mechanisms for inhibition^7,9,10^.

Neem modulates carcinogenic metabolizing enzymes, improving antioxidant status of the body and inhibits the synthesis of essential metabolites and prostaglandins^11^. Many of them are responsible for tumor production. Further, it was found that the plant extract inhibits P-450 monooxygenase system and down regulate p-21 ras oncogene. The p21 ras oncogene is a proto-oncogene that encodes the Ras protein which regulate cell growth, proliferation, and survival. When ras genes mutated, they become oncogenic and lead to uncontrolled cell division and tumorigenesis^12,13^. These properties require more investigation before pre-clinical involvement. This study aimed to assess the antioxidant properties of the leaf extract by measuring SOD and CAT activities, as well as evaluating its total antioxidant capacity and DPPH-scavenging potential. The extract’s effect on the growth of A549 and MCF-7 cells was also investigated, along with possible mitochondrial-mediated mechanisms. Cell-cycle progression was analyzed using flow cytometry to identify phase-specific changes, and qRT-PCR was performed to examine the extract’s impact on genes involved in apoptosis and cell survival. In addition, the antimicrobial activity of the extract was evaluated.

## Materials and methods

### Collection and processing the plant material

*Azadirachta indica* A. Juss commonly known as neem, nimtree or Indian lilac, is a tree in the mahogany family Meliaceae. It is native to the Indian subcontinent and most of the countries in Africa. It is typically grown in tropical and semi-tropical regions. Neem trees also grow on islands in southern Iran. Its fruits and seeds are the source of neem oil.

The leaves are collected from Capital Central Park (El-Hadaeq Al-Markazeya), located within the New Administrative Capital to the east of Cairo, Egypt (30.011563° N, 31.654947° E) during the autumn season (October 8th, 2023). This park forms an elongated urban green corridor established on reclaimed desert land and bordered by recently developed residential and governmental districts. The site lies in a hot desert climatic zone (Köppen BWh), with very long, dry and hot summers, short mild winters, and extremely low annual rainfall. The native substrate consists of weakly calcareous sandy to sandy-loam desert soils that originally supported sparse natural vegetation but have been reshaped into an irrigated landscaped area with lawns, ornamental shrubs, and regularly spaced shade trees, including A. indica planted along internal pedestrian routes and service roads.

 A voucher specimen was deposited at the Department of botany and microbiology, Faculty of Science, Herbarium, Damietta University (DAM000023).

Firstly, the leaves were dried in shade until complete dryness. The leaves branches were removed and only the leaves were ground into a powder.

### Preparation of the neem leaves crude extract (NLE)

The powdered plant material (30 g) was soaked in approximately 1 L of absolute ethanol at room temperature, protected from light, under continuous stirring overnight. After this period, the extract was filtered to separate the solid material from the ethanolic extract. The filtrate of the crude leaf extract was then dried in a hot oven at 50 °C for 12 h to obtain a viscous honey-like crude extract. After this process, the crude extract was then stored at -5 °C.

### Determination of total phenolic components (HPLC)

An Agilent (1260) series was used to determine the total phenolic components. The Zorbax Eclipse Plus C8 column (46 cm x 2500 cm) was used to perform the separation. Water and 0.05% trifluoroacetic acid in acetonitrile at a flow rate of 0.9 ml/min made up the mobile phase. In a linear gradient, the mobile phase was programmed as the method of Khalil et al.^14^.

### Determination of the antimicrobial activities

#### Antimicrobial activities

Using agar well diffusion method, 0.15 mg/ml of the neem extract were dissolved in ethyl alcohol and evaluated as antimicrobial agents^15,16^. A solvent control, consisting of ethyl alcohol alone at a maximum concentration of 1% (v/v), was also included in all assays and showed no antimicrobial activity against any of the tested microorganisms. The antimicrobial activities were determined towards bacterial and yeast strains that obtained from the Microbiology Laboratory, Faculty of Science, Damietta University, Egypt. Gram-positive bacteria (*B. cereus* HES3 OR553494 and *Staphylococcus epidermidis* ATCC 12228), Gram-negative bacteria (*E. coli* D8 MF062579 and *Enterobacter aerogenes* ATCC 13048), and yeast (*Candida albicans* ATCC 10231) were sub cultured on nutrient agar and yeast extract-peptone-dextrose (YEPD) agar media, respectively. Using the spread plate method, the examined microorganisms were inoculated (0.5 McFarland standard) after the culture media had been prepared, autoclaved, and put into sterile Petri plates. The tested extract (150 µg/ml) was aseptically applied in 100 µl portions to 5 mm perforated wells. After 24 h., the inhibition zones were determined and expressed in millimeters. The prescription drugs fluconazole and tetracycline were included as positive controls; each tested at a fixed concentration of 150 µg/ml to assess their maximum potential inhibitory activity against the test organisms.

#### Minimum inhibition concentration (MIC)

Using the serial dilution method, MIC of neem extract was evaluated. The extract was dissolved at different concentrations ranging from 5 to 50 µg/ml. Nutrient broth and YEPD broth medium was prepared and inoculated by 0.5 MacFarland of bacterial and yeast strains, respectively. After incubation at 37 °C for 24 h or at 30 °C for 48 h for bacterial and yeast strains, respectively. A spectrophotometric analysis was measured at 600 nm to assess the microbial proliferation^17^.

#### The enzymatic antioxidant activity

The Whittenbury^18^ and Sakamoto and Komagata^19^ methods were used to measure the peroxidase (POX) and catalase (CAT) activities for microbial strains treated with extract. For the enzymatic reaction, 4-aminoantipyrine, potassium phosphate buffer, 2,4-DCP, and H_2_O_2_ were mixed with the extract’s MIC for a minute in order to gauge POX activity. To measure CAT activity, extract MIC was mixed with phosphate buffer and H_2_O_2_. Using spectrophotometry at 510 and 415 nm, the oxidation-induced increase in absorbance was assessed in relation to the untreated microbes as a control. The number of moles of H_2_O_2_ per liter was one-unit mL^− 1^ of enzyme^20^.

### Determination antioxidants activities

#### 2,2^2^-diphenyl-1-picrylhydrazyl (DPPH) scavenging activity

Scavenging activity of DPPH was done according to the method of Algfri et al.^21^ with some modification.

0.1 mM DPPH solution was prepared in methyl alcohol. Various amounts of the plant extract 10 mg/ml (1–10 µl) were mixed with DMSO to reach 20 µl, and then 1.48 ml of DPPH solution was added. The reaction mixture was incubated at room temperature in dark for twenty minutes. The absorbance of the prepared mixture was measured at 517 nm. Two milliliters of DPPH solution were used as a control. The extract’s scavenging radical activity was determined using the equation below.

$$\:\mathbf{\%}\:\mathbf{R}\mathbf{a}\mathbf{d}\mathbf{i}\mathbf{c}\mathbf{a}\mathbf{l}\mathbf{s}\:\mathbf{S}\mathbf{c}\mathbf{a}\mathbf{v}\mathbf{e}\mathbf{n}\mathbf{g}\mathbf{i}\mathbf{n}\mathbf{g}\:\mathbf{A}\mathbf{c}\mathbf{t}\mathbf{i}\mathbf{v}\mathbf{i}\mathbf{t}\mathbf{y}\:\left(\mathbf{R}\mathbf{S}\mathbf{A}\right)=\frac{\boldsymbol{A}\boldsymbol{c}-\boldsymbol{A}\boldsymbol{s}}{\boldsymbol{A}\boldsymbol{c}}\boldsymbol{*}100$$

Where **Ac** is the control absorbance and **As** is the Sample mixture absorbance.

#### Superoxide dismutase (SOD)- like activity

The SOD like activity of the extract was estimated by the method of Dechatelet et al.^22^.

$$\begin{gathered} {\mathbf{Percent}}{\text{ }}{\mathbf{of}}{\text{ }}{\mathbf{inhibition}}{\text{ }}{\mathbf{reduction}}{\text{ }}{\mathbf{of}}{\text{ }}{\mathbf{nitro}}{\text{ }}{\mathbf{blue}}{\text{ }}{\mathbf{dye}} \hfill \\ \;\; = {\raise0.7ex\hbox{${\left( \begin{gathered} \user2{rate~of~the~change~of~blank~} \hfill \\ - \user2{rate~of~the~change~of~~the~sample~} \hfill \\ \end{gathered} \right)}$} \!\mathord{\left/ {\vphantom {{\left( \begin{gathered} \user2{rate~of~the~change~of~blank~} \hfill \\ - \user2{rate~of~the~change~of~~the~sample~} \hfill \\ \end{gathered} \right)} {\left( {\user2{rate~of~the~change~of~blank}} \right)\user2{~}}}}\right.\kern-\nulldelimiterspace} \!\lower0.7ex\hbox{${\left( {\user2{rate~of~the~change~of~blank}} \right)\user2{~}}$}}*{\mathbf{100}} \hfill \\ \end{gathered}$$

#### Catalase like activity

The extract’s catalase-like activity was determined using this method of Sinha^23^.

$${\mathbf{Catalase}}{\text{ }}\left( {{\mathbf{U}}/{\mathbf{L}}} \right)={\mathbf{1}}/{\mathbf{t}}{\text{ }}*{\mathbf{log}}{\text{ }}{{\mathbf{S}}_{\mathbf{0}}}/{\mathbf{S}}$$

Where **S**_**0**_is H_2_O_2_ initial concentration, **S** is H_2_O_2_ concentration at **(t)** mins.

#### Total antioxidants capacity (TAC) of the ethanolic neem extract

The Total antioxidants capacity of the plant extract was estimated following to the method of Koracevic et al.^24^.

$${\mathbf{TAC}}{\text{ }}\left( {{\mathbf{mM}}/{\mathbf{L}}} \right) = {\mathbf{C}}_{{{\mathbf{uA}}}} {\mathbf{X}}\left( {{\mathbf{K}} - {\mathbf{A}}} \right)/\left( {{\mathbf{K}} - {\mathbf{UA}}} \right)$$

K: the control absorbance

A: sample absorbance

UA: standard uric acid absorbance

C_UA_: uric acid concentration

### Cytotoxicity of neem extract on different cell lines

The mitochondrial dependent reduction of yellow MTT (3-(4,5-dimethylthiazol-2-yl)-2,5-diphenyl tetrazolium bromide) to purple formazan^25^ was used to evaluate the cell viability. The MTT reagent was purchased from Sigma-Aldrich, St. Louis, MO, USA. A stock solution was prepared at 5 mg/mL in sterile phosphate-buffered saline (PBS) and passed through a 0.22 μm syringe filter to ensure sterility. For the cytotoxicity assay, the working solution was added to the cells at a final concentration of 2.5 µg/mL.

All experimental procedures were conducted under sterile conditions within a Class II biosafety laminar flow cabinet (Baker, SG403INT, Sanford, ME, USA). The MCF-7 human breast cancer cell line, the A549 lung carcinoma cell line and BNL mouse normal liver cells were cultured in DMEM/F-12 medium supplemented with 1% L-glutamine and 1% antibiotic-antimycotic solution containing 10,000 U/mL penicillin, 10,000 µg/mL streptomycin sulfate, and 25 µg/mL amphotericin B. Cells were maintained at 37 °C in a humidified atmosphere with 5% CO₂ using a water-jacketed CO₂ incubator.

After 10 days of batch culturing, the cells were seeded at a density of 10 × 10³ cells per well into 96-well microplates containing complete growth medium and incubated for 24 h under standard conditions (37 °C, 5% CO₂). The medium was then replaced with serum-free medium to prevent any interactions between serum proteins and the extract, allowing an accurate evaluation of its cytotoxic effect, and the cells were treated with a range of concentrations of the test extract (1000, 500, 250, 125, 62.5, 31.25, 15.6, and 7.8 µg/mL) which was dissolved in DMSO to make a stock solution, and was further diluted in serum-free medium to achieve the desired test concentrations. The final DMSO concentration in the culture wells did not exceed 0.2% to avoid any solvent-induced cytotoxicity. For each concentration of the neem extract, the assay was performed in three wells (technical replicates) within the same experiment. To ensure reproducibility, the entire experiment was independently repeated three times using freshly cultured cells (biological replicates).

Following a 48-hour incubation, the medium was carefully removed, and each well was supplemented with 40 µL of MTT solution at a final concentration of 2.5 µg/mL. The plates were incubated for an additional 4 h under the same conditions. Then 200 µL of 10% sodium dodecyl sulfate (SDS) in deionized water was added to each well, and the plates were left overnight at 37 °C.

As a positive control, 100 µg/mL of a known cytotoxic natural compound (doxorubicin) was used under identical conditions to ensure total cell death, as described by Thabrew et al.^26^.

The following formula was used to determine the percentage change in viability.

$$\left( {\left( {{\mathbf{Reading}}{\text{ }}{\mathbf{of}}{\text{ }}{\mathbf{extract}}{\text{ }}/{\text{ }}{\mathbf{Reading}}{\text{ }}{\mathbf{of}}{\text{ }}{\mathbf{negative}}{\text{ }}{\mathbf{control}}} \right){\text{ }} - {\mathbf{1}}} \right){\text{ }}{\mathbf{x}}{\text{ }}{\mathbf{100}}$$

### Cell cycle analysis

Cell cycle analysis was performed using flow cytometry to assess potential alterations in the distribution of cell cycle phases between treated and untreated A549 lung cancer cells. Three experimental groups were prepared, each containing 1 × 10⁶ A549 cells. The first group served as the negative control (untreated), the second was treated with doxorubicin as a positive control, and the third received neem extract at its IC₅₀ (128 µg/ml) concentration.

Cells from each group were suspended in 0.5 mL of 1× Dulbecco’s Phosphate-Buffered Saline (DPBS) and gently aspirated several times using a Pasteur pipette. The cell suspensions were then fixed by adding 70% ethanol and incubated on ice for 2 h. Following fixation, cells were centrifuged, and the ethanol supernatant was carefully removed. The resulting pellets were resuspended in 5 mL of 1× DPBS for 30 s, then centrifuged. After washing, each pellet was resuspended in 1 mL of propidium iodide (PI) staining solution and incubated at room temperature in dark for 30 min. The distribution of cells across the G0/G1, S, and G2/M phases of the cell cycle was analyzed using CytExpert software^27^.

### Quantitative real-time PCR (qRT-PCR)

Quantitative real-time PCR (qRT-PCR) was conducted to evaluate gene expression changes in A549 cells treated with neem leaf extract. A549 cells were seeded in 6-well plates at a concentration of 4 × 10⁵ cells/mL and exposed for 24 h to 128 µg/mL of neem extract (IC_50_ determined by MTT assay, this concentration was selected because it allows around 50% of the cells to remain viable, providing sufficient living cells for reliable measurement of apoptotic and anti-apoptotic gene expression, while avoiding excessive cell death that could compromise RNA integrity). Total RNA was isolated from both treated and untreated (control) cells using TRIzol reagent, following the manufacturer’s protocol. Subsequently, 1 µg of total RNA from each sample was reverse-transcribed into complementary DNA (cDNA) using the Bio-Rad SYBR Green PCR Master Mix kit. The qRT-PCR reactions were carried out on a Rotor-Gene real-time PCR system (Corbett Research, Sydney, Australia) using gene-specific primers for BAX, BCL2, and P53^28^.

### Statistical analysis

Data were analyzed using SPSS software (version 22). All results were expressed as mean ± STD. Differences in means between the various treated cells or media and control were analyzed by one-way ANOVA test and the results were considered statistically significant if p ˂0.05.

## Results

### Total phenolic compounds of the neem extract

**Fig. 1:**
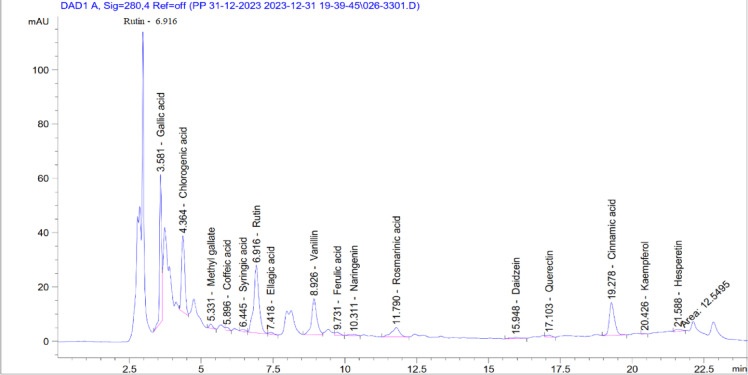
Distribution of the phenolic compounds of the neem leaves extract.

The name of each phenolic compounds and its content percent in the used neem leaves extract are listed in Tables 1and their separation pattern is depicted in Fig. 1.

**Table 1:** Type and Percentages of phenolic compounds in the extract.

Component	Conc. (µg/g)
Rutin	2345.18
Chlorogenic acid	1451.47
Gallic acid	1217.34
Rosmarinic acid	367.16
Vanillin	310.67
Cinnamic acid	136.93
Querectin	61.56
Naringenin	40.99
Ellagic acid	37.79
Syringic acid	36.71
Ferulic acid	33.59
Coffeic acid	32.45
Methyl gallate	31.02
Hesperetin	30.85
Daidzein	26.98
Kaempferol	7.50

### Antimicrobial activities

The antimicrobial activity of neem leaves extract was tested against different Gram-positive (*Bacillus cereus* and *Streptococcus epidermidis)*, Gram-negative bacteria *(Escherichia coli* and *Enterobacter aerogenes)* and as a fungus, yeast (*Candida albicans*) is also used. As a standard antibacterial drug, Tetracycline was used but fluconazole was used as a standard antifungal drug.

#### Agar well diffusion method

The zones of inhibition (mm) for each microorganism treated with the extract or standard drugs were shown in Tables 2 and 3. The inhibition zones of the prepared extract showed that it is more effective against Gram-negative bacteria than Gram-positive one. The highest antibacterial action of the extract was against *E. aerogenes* compared to both *B. cereus* and *S. epidermis* that displayed the smallest inhibition zones. Further, it was notable that the extract had a stronger antibacterial potential against the tested Gram-negative bacterial strains compared to the well-known standard antibacterial; namely, tetracycline. Further, the extract revealed an antifungal action against the tested yeast with an inhibition zone of 16 mm which was similar to that of the standard antifungal drug; namely, fluconazole (15 mm).

**Table 2:** Antimicrobial activities of the extract against Gram-positive and Gram-positive bacteria versus the standard antibiotic (Tetracycline) and a standard antifungal drug (Fluconazole).

Compound	Gram-positive bacteria		Gram-negative bacteria		yeast
	Bacillus cereus	Streptococcus epidermidis	Escherichia coli	Enterobacter aerogenes	Candida albicans
Neem extract	13 ± 0.06^*^	13 ± 0.03^***^	16 ± 0.14^***^	23 ± 0.06^***^	16 ± 0.14^**n**^
Tetracycline	14 ± 0.05	18 ± 0.06	10 ± 0.14	12 ± 0.03	**-**
Fluconazole	**-**	**-**	**-**	**-**	15 ± 0.07

Data are represented for neem extract compared to a standard antibiotic (Tetracycline) and a standard antifungal drug (Fluconazole). Asterisks (*, **, ***) indicates low significant, significant and highly significant, respectively while (n) not significant.

**Table 3:** Agar well diffusion method test of the tested neem extract against Gram-positive and Gram-negative bacteria as well as yeast as a fungal example.

Compound	Gram-positive bacteria		Gram-negative bacteria		Yeast
	*Bacillus cereus*	*Streptococcus epidermidis*	*Escherichia coli*	*Enterobacter aerogenes*	*Candida albicans*
Neem extract					
Tetracycline					
Fluconazole					

#### Minimum inhibition concentration (MIC)

**Fig. 2:**
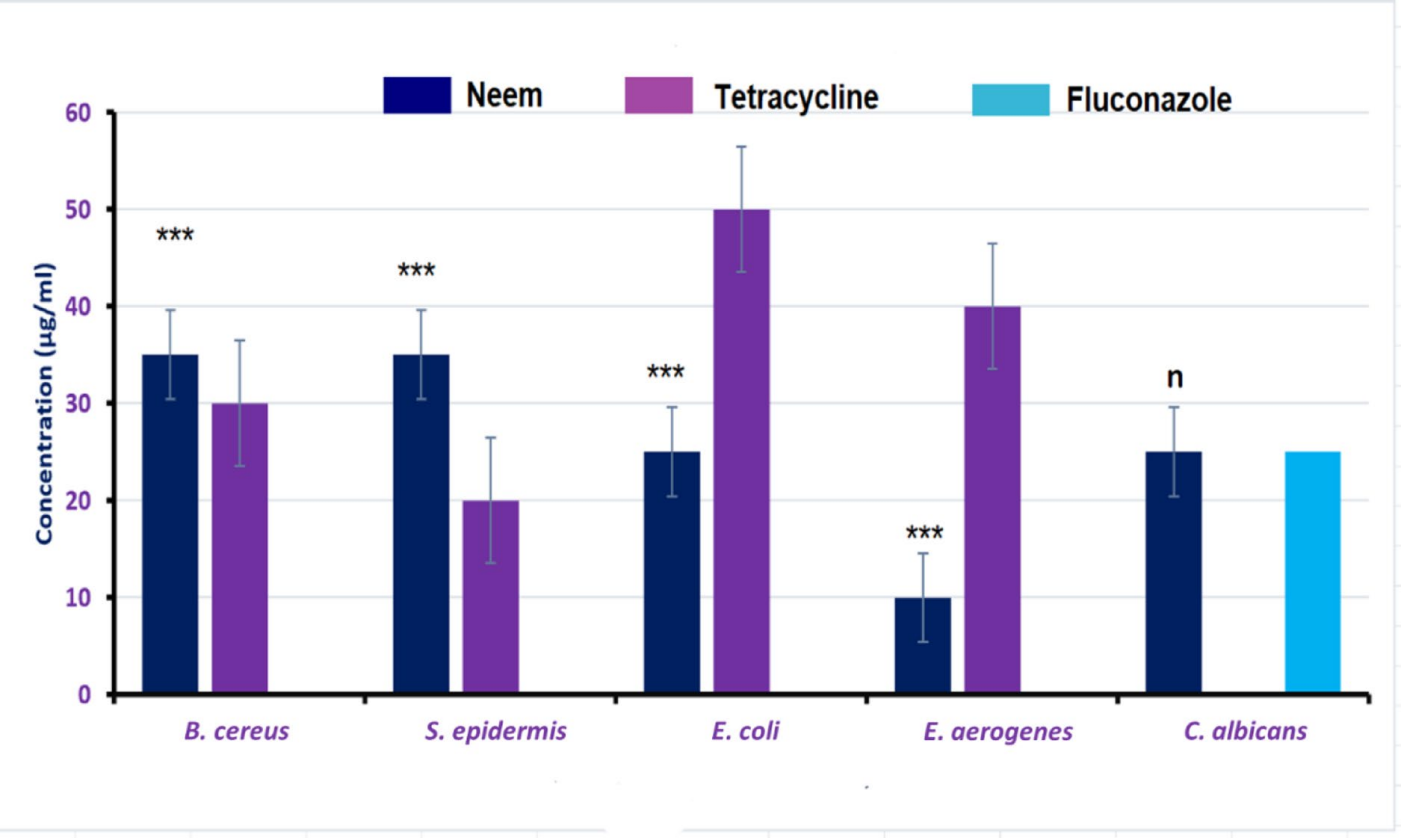
Minimum inhibition concentration test of the neem extract against Gram-positive and Gram-negative bacteria versus a standard antibiotic (Tetracycline) and a standard antifungal drug (Fluconazole). Asterisks (***) indicates highly significant while (n) represent non-significant.

The results of the minimum inhibition concentrations (the minimum amount of the extract that, after an overnight incubation with media, stops a microorganism from growing visibly) of neem leaves extract towards the selected microorganisms versus that of tetracycline as antibacterial drug and fluconazole as antifungal one were shown in Fig. 2.

#### The enzymatic antioxidants activities

**Fig. 3:**
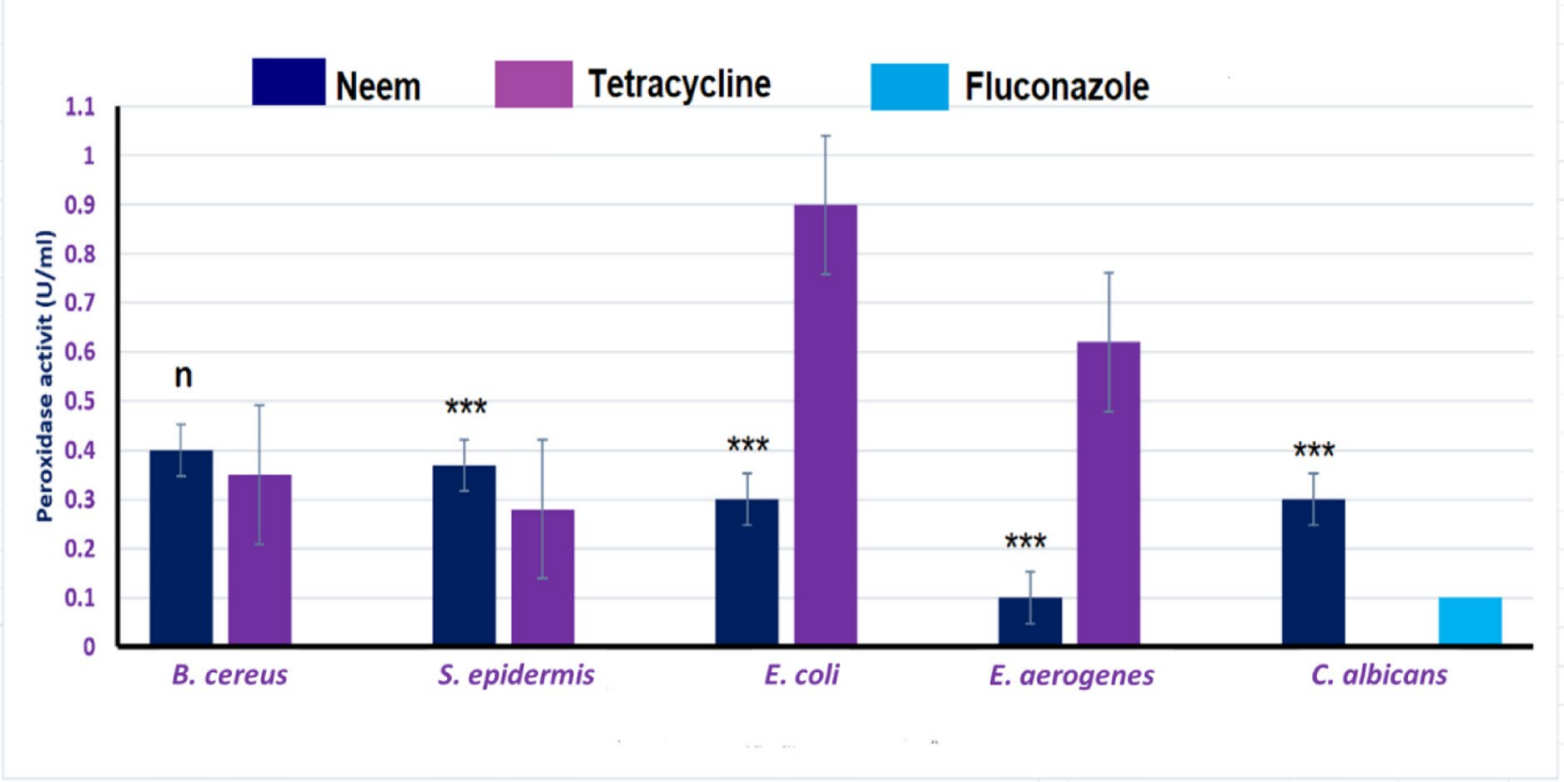
Effect of neem extract on the peroxidase activity of the selected microorganisms. Data are represented for neem when compared to tetracycline and fluconazole. Asterisks (***) indicates highly significant, while (n) is not significant.

**Fig. 4:**
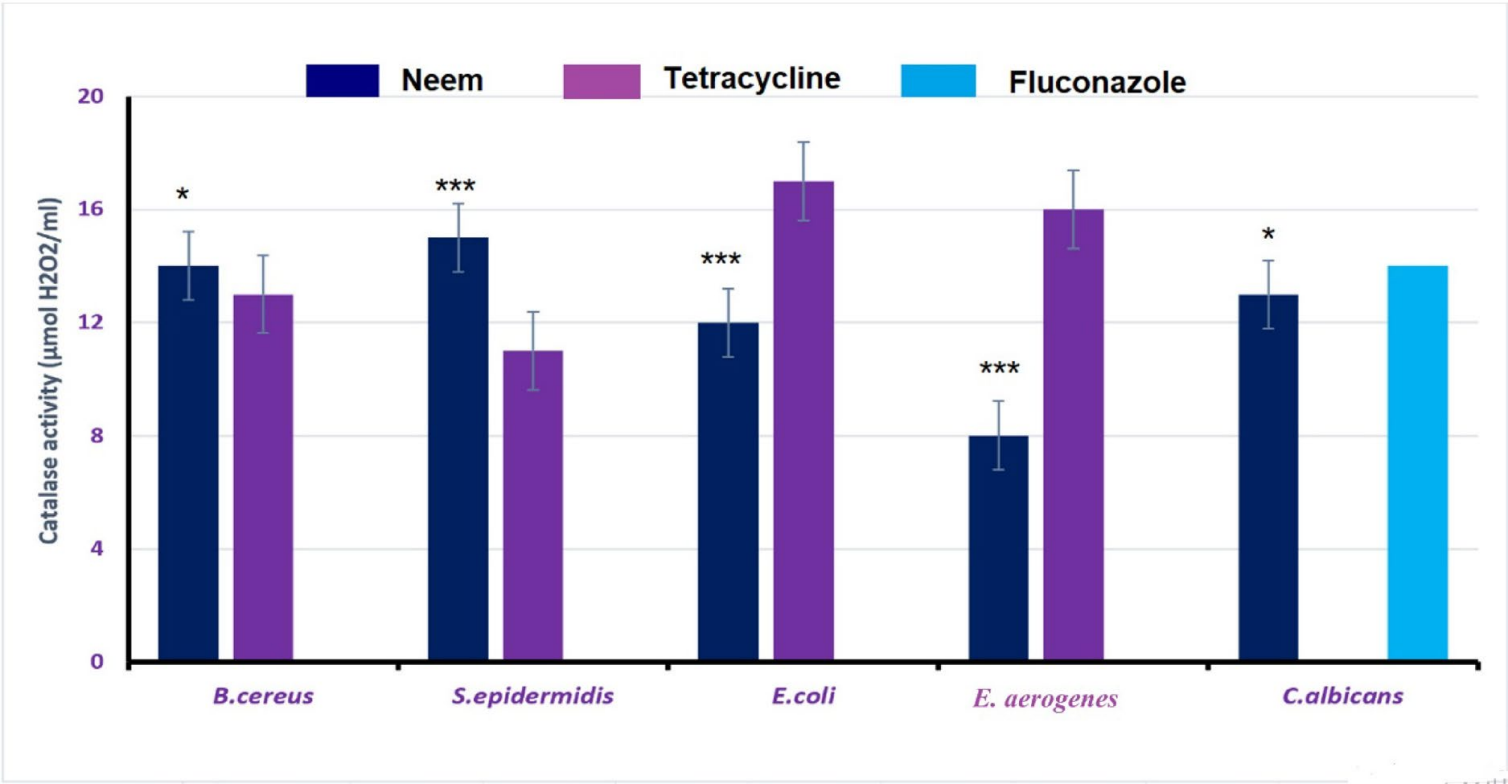
Effect of neem extract on the catalase activity of the selected microorganisms. Data are represented for neem compared to tetracycline and fluconazole. Asterisks (*, ***) indicates low significant and highly significant, respectively.

The effect of treating of different microorganisms with neem leaves extract on their enzymatic antioxidant activities (peroxidase and catalase) were shown in Figs. 3 and 4. The results showed that, the extract significantly decreased the activities of both peroxidase and catalase enzymes in Gram-negative bacterial strains while it was non-effective for those of Gram-positive ones if compared to the tetracycline values. Further, for *C. albicans*, the extract showed low significant decrease in catalase activity but it was unable to decrease peroxidase activity if compared to fluconazole.

### Antioxidant activities of neem extract

The antioxidant activities of the characterized neem leave extract were tested with different assays versus ascorbic acid (as a positive control). Their results are listed in Table 4. The results showed that; neem extract has a highly and significantly scavenging capacity for free radicals when compared to that of ascorbic acid. This is due to it had a much lower DPPH IC_50_ than that of ascorbic acid. Further, the extract has the ability to scavenge H_2_O_2_ radicals and inhibits the phenazine methosulphate (PMS) mediated reduction of nitroblue tetrazolium salt (NBT) in the catalase (CAT) and superoxide dismutase (SOD)- like activities, respectively. Additionally, it has a highly significant total antioxidant capacity (TAC) versus vitamin C control.

**Table 4:** Antioxidant activities of neem extract.

Antioxidants assay	Ascorbic acid (positive control)	Neem leaves extract (test sample)
DPPH (IC_50_%)	0.51 ± 0.0.12 mg/ml	0.225 ± 0.0102^***^mg/ml
SOD (Inhibition %)	54.2 ± 5.2%	68 ± 2.3%^***^
CAT (U/l)	4.3 ± 0.12	8.3 ± 0.21^***^
TAC (mM/l)	1.12 ± 0.03	1.625 ± 0.026^***^

1. The calculated activities of SOD, CAT and TAC were determined at concentration of 10 mg/ml of both extract and ascorbic acid.

2. Data represent the comparison between neem and vitamin C. Asterisks (***) indicates highly significant.

### Cytotoxic effects on human cell lines

**Fig. 5:**
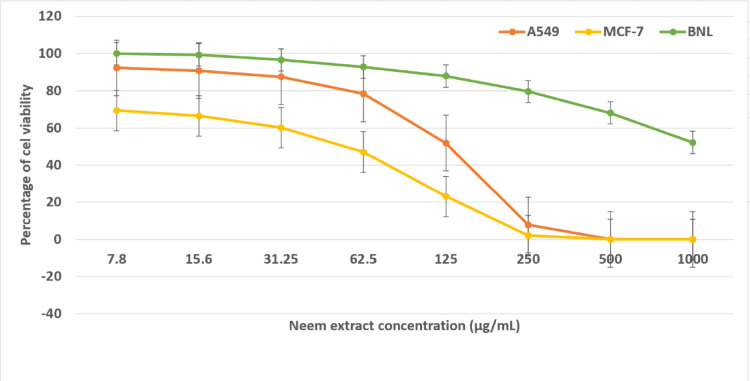
Cytotoxic effects of neem extract on A549, MCF-7 and BNL cell lines after incubation for 48 h.

The cytotoxicities of neem leaves extract against A549, MCF-7 and BNL cell lines were determined using MTT assay. The results of these cytotoxicities are depicted in Fig. 5. The results indicated that, the neem leaves extract at concentrations ranging from 7.8 to 1000 µg/mL caused a decrease in the cells viabilities of the A549 and MCF-7 cell lines at IC_50_ of 128.1 and 55.7 µg/mL for these cell lines, respectively. Furthermore, by increasing the neem dose of extract (equivalent to 237 and 176.µg/mL), 90% of cells viabilities for A549 and MCF-7 cell lines, respectively were lost. On the other hand, the neem leaf extract showed little effect on normal mouse liver cells at lower concentrations, and even at the highest concentration tested it did not reduce cell viability by 50%. Generally, the results proved that the extract was more toxic to MCF-7 cell line than that of A549 one.

### Cell cycle analysis

**Fig. 6:**
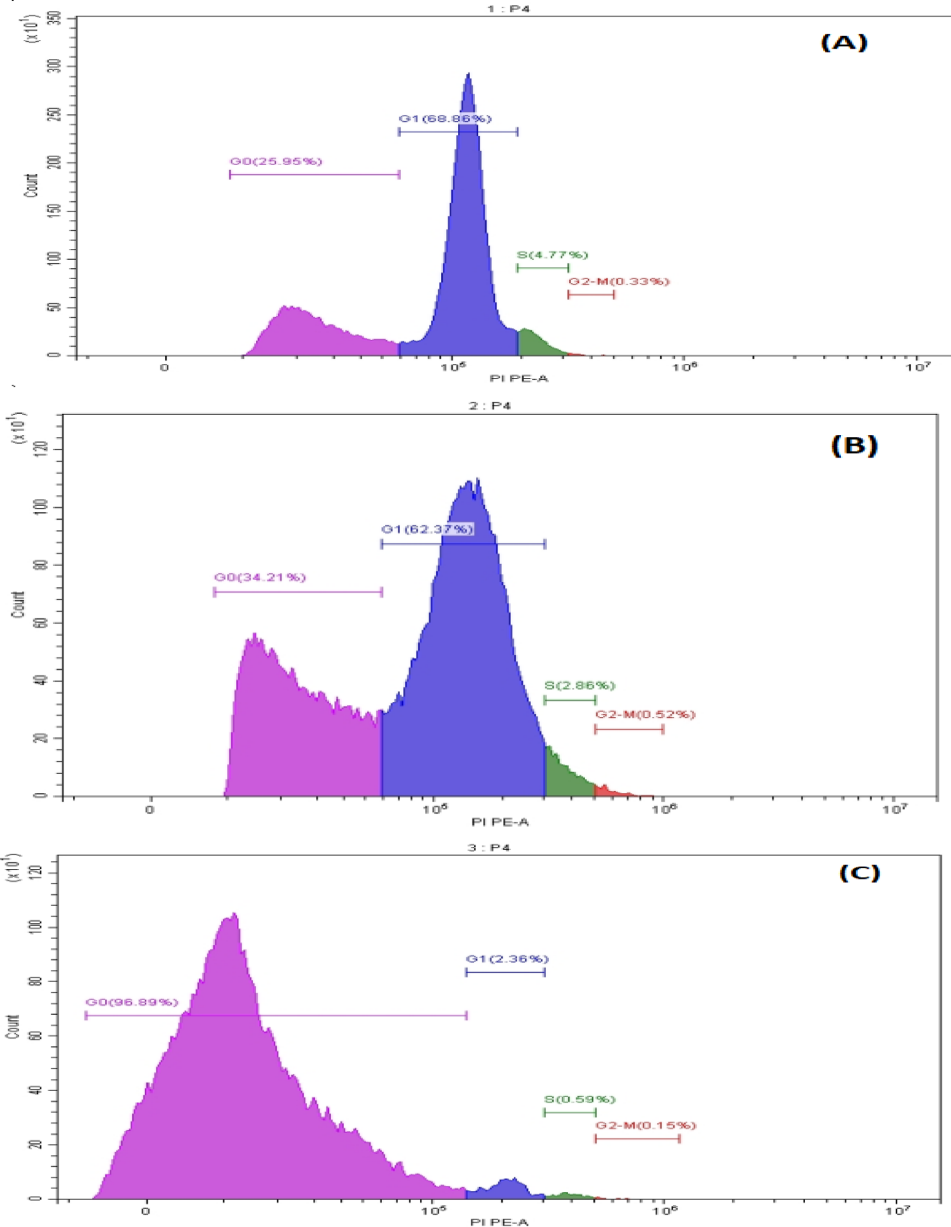
Flow cytometric chart of the analysis of the cells cycle of the negative control s (**A**), positive control (doxorubicin- treated cells, (**B**) and those of the neem extract-treated cells (**C**).

A549 cell line was used to determine the effects of neem extract on the parameters of these cell cycles in vitro. A flowcytometry technique was used to measure the distribution of cells population among the different stages of the cell cycle depend on the content of DNA after PI staining. The Flowcytometric charts plotting of these results were as shown in Fig. 6. After treating the A549 cell line with the neem extract at IC_50_ concentration, most of the cells (96.89%) accumulated in the G0 phase (arrest phase) which is a highly significant compared to both negative (non-treated cell) and positive control (cells which were treated with doxorubicin as a standard drug). While, the cell percentage in G1 stage was only 2.36%, with a decrease in the cells number in the S and G2-M stages (0.56 and 0.15%, respectively) (Table 5).

**Table 5:** Percent of populations of negative (untreated cells) and positive control (doxorubicin treated cells) as well as neem-extract treated cells.

Population	Events (cell)			Percent (%)		
	Negative control	Positive control	Neem treated cells	Negative control	Positive control	Neem treated cells
G0	12,957	17,101	42,625^***^	25.95	34.21	96.89^***^
S	2380	1429	258^***^	4.77	2.86	0.59^***^
G2/M	166	260	68^***^	0.33	0.52	0.15^***^
G1	34,380	31,177	1040^***^	68.86	62.37	2.36^***^

Data are represented for neem compared to negative and positive controls. Asterisks (***) indicates highly significant.

### Effects of neem leaves extract on apoptosis-related genes expression of A549 Cells

**Fig. 7:**
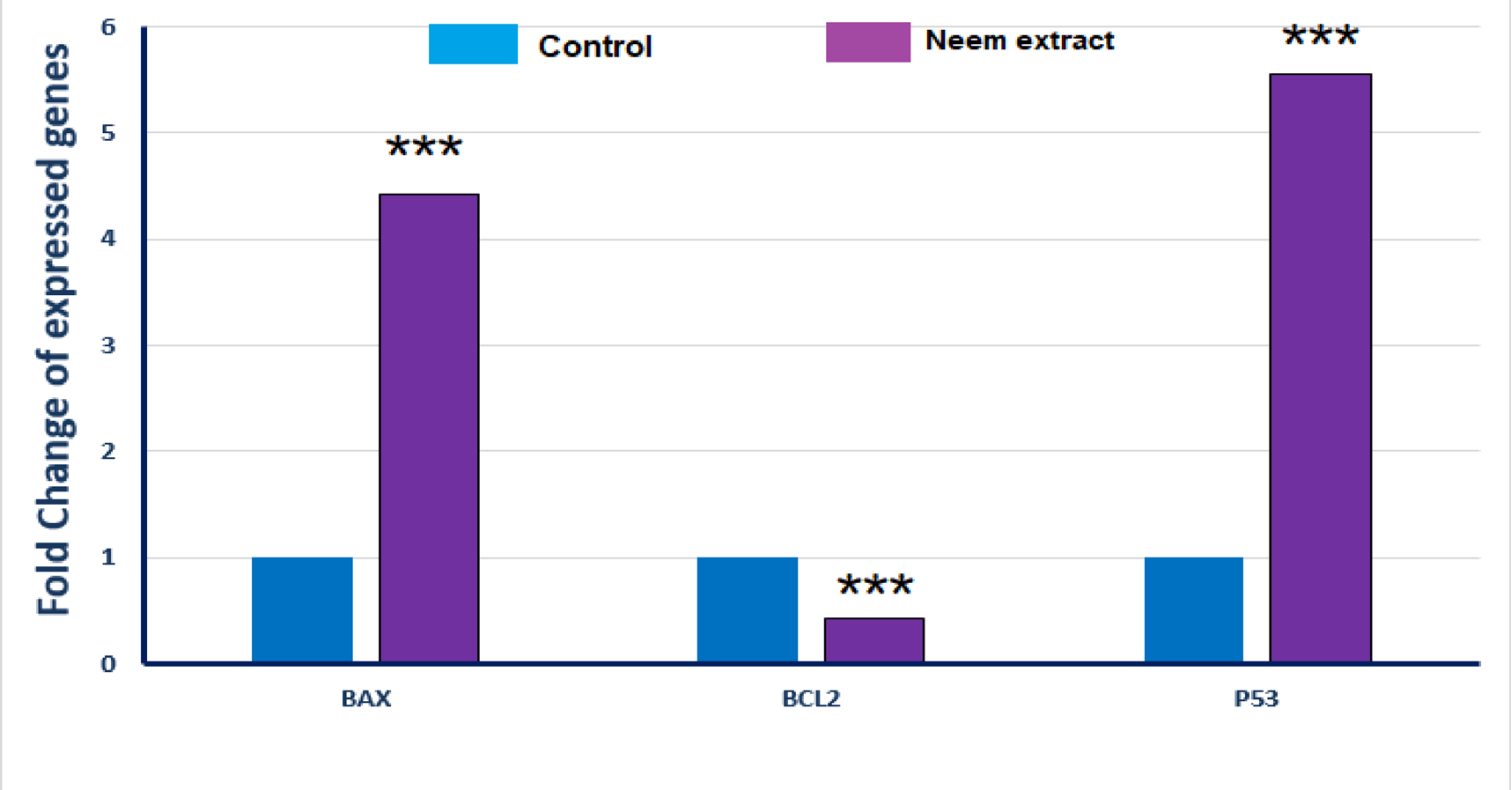
The effects of ethanolic extract of neem leaves on the expression of the pro-apoptotic and the anti-apoptotic genes in A549 cell line. Data are represented for neem compared to control cells. Asterisks (***) indicates highly significant differences.

In this regard, qRT-PCR analysis was done to measure the amount of three apoptotic genes; namely BAX, BcL-2 and P53 in the extract-treated A549 cells at 128 µg/mL(IC_50_). The results of this technique was shown in Fig. 7. A considerable reduction in the anti-apoptotic BcL-2 gene (0.58% of the control) was recorded, while the pro-apoptotic genes P53 and BAX expression was increased (555% and 442% of that of the control).

## Discussion

Polyphenolic compounds play a significant role in antioxidant, antimicrobial as well as anticancer^29^. Total polyphenolic compounds screening of the ethanolic neem leaves extract in the current study revealed the presence of high amounts of rutin (2.35 mg/g), gallic acid (1.21 mg/g) and chlorogenic acid (1.41 mg/g). These polyphenolic compounds were proposed to possess anticancer, antibacterial, antiviral, antioxidant, and anti-inflammatory properties.

Similar result was obtained by Sarmiento et al.^30^ in which the major polyphenolic compounds presented in the neem leaves extract was rutin (4.17 mg/g), chlorogenic acid (0.9 mg/g) and gallic acid (0.32 mg/g). Also, their results proved that the total phenolic compounds of the70% ethanolic neem extract was 11.87 mg /g. These phenolic compounds have an important role against progress of malignant cells as they are able to suppress the tumor proliferation, stimulate apoptosis and arrest the cell cycle. They also have a great antioxidant and antimicrobial activity as well as immunomodulatory effects^30^.

The antimicrobial activity of ethanolic extract of neem leaves was studied on different microorganisms in this study. The results proved that the antibacterial activity of neem leaves extract was more effective towards Gram-negative bacteria than those of Gram-positive ones when compared to the tetracycline (positive control). This is because MIC needed to inhibit Gram-negative ones was significantly lower than that of Gram-positive ones.

Also, by studying the enzymatic antioxidants activities of these bacteria we found that, treatment of these bacteria with neem leaves extract inhibited peroxidase and catalase enzymes compared to the tetracycline treated bacteria. In detail, the effect of our extract was more significant towards Gram-negative bacteria than the Gram-positive ones compared to tetracycline.

For *C. albicans* yeast, the antifungal effect of neem extract was similar to that of fluconazole one as they had the same MIC and closely values of peroxidase and catalase activities. We can suggest that, the antimicrobial activities of neem are due to its phytochemicals contents which mediate such antimicrobial effects against the listed microorganisms.

Similar results were represented by Ahmed et al.^31^ as they found that neem extract showed antibacterial activity towards *E. aerogenes* at 50 mg/mL with an inhibition zone of 9.4 ± 1.13 mm. Our results were also similar to that of Mudenda et al.^32^ as the MIC for their ethanolic extracts of neem was 20 mg/ml against *E. coli*. As well as the results of Francine et al.(2015) and ogidi et al.^33,34^ in which the inhibition zone of *E. Coli* was only 10.0 ± 1.0 mm for methanol neem extract. The studies of Alzohairy et al.(2016) and Hemdan et al.^35,36^ showed that 50 µg/ml of neem extract were able to inhibit a wide-spectrum of the tested Gram-negative bacteria as *E. coli* (MIC = 50 mg/mL within 30 min), *S. enterica* (MIC = 25 mg/mL within 30 min) and *E. aerogenes* (MIC = 25 mg/mL). Finally, the study of Mudhafar et al.^37^ showed that the ethanolic neem extract gave remarkable efficacy towards several foodborne pathogenic bacteria, with levels of activity ranging from 20 µg/ml to 350 µg/ml. The differences in results may be due to species difference of bacteria or the source of neem leaves.

Many plants show antioxidant activities owing to their flavonoids and phenolics contents^38^. The ethanolic neem extract were found to be biologically active and possess strong antioxidant and therapeutic activity^39^. This is mainly due to polyphenolic compounds contents which were quantitatively estimated in HPLC.

In this study, the antioxidant activity of neem leaves was estimated using the DPPH, SOD and CAT like activity as well as TAC capacity. After estimating the last for parameters, the plant’ extract showed a highly significant antioxidant effect compared to that of ascorbic acid as a positive control. The neem leaves extract was able to scavenge the free radicles of DPPH, inhibit the phenazine methosulphate mediated reduction of NBT as well as convert hydrogen peroxide into molecular oxygen and water in catalase like mechanism. Finally, the TAC of the neem extract was measured versus ascorbic acid as a control. The results were high significant compared to those of the ascorbic acid.

These results are similar to that of Nasr et al.^40^ in which neem leaves extract showed a strong antioxidant activity based on 50.6% reduction in malondialdehyde (MDA) level. Also, the results obtained by Hafeez et al.^41^ showed that neem extract had a moderate antioxidant activity based on the reduction in the residual DPPH free radical. This is because 59.06% of these radicals were still present in the radical form when 250 µg/ml of the extract was added. Additionally, the study of Mudhafar et al.^37^ showed that, the DPPH scavenging free radical percent ranged from 44.15% (at 200 µg/ml) and 80.10% (at 1000 µg/ml) with an IC_50_ value of 281.03 µg/ml for ethanolic extract. Therefore, the antioxidant activity of neem extract is the cause of stopping the oxidation process in the human body after its exposure to either free radical attacking or those produced normally from chain reactions. This chain reaction may cause cancer via damage the cell organelles especially after reduction or consumption of endogenous antioxidants. Since antioxidants able to terminate these long chains of free radicals, one can expect that neem treatment will be the case^4,42^.

In this study, the cytotoxic effect of neem leaves extract was examined on two different cell lines; namely MCF-7 and A549. The results showed that MCF-7 cells were more sensitive towards the extract of neem leaves than A549 cells as the IC_50_ of MCF-7 and A549 cells were 55.7 and 129.1 µg/ml, respectively.

Triple et al.^43^ studied the cytotoxic effect of neem extract on MCF-7 and HeLa cells, their results indicated that the extract was more toxic to MCF-7 than HeLa cells. Also, the study of Santos et al.^44^ showed that, the neem extract had a maximum cytotoxicity of 68% at 500 µg/ml against A549 lung cancer cells and minimum cytotoxicity of 12% at 50 µg/ml. While, in the current study a 90% growth inhibition of such cells was observed at only 237 µg/ml.

According to the criteria introduced by the National Cancer Institute for screening crude plant extracts, samples with IC₅₀ values below roughly 30 µg/mL are regarded as strongly active against cancer cells, while higher values indicate weaker effects. This guideline originates from the work of Suffness and Pezzuto^45^, who outlined the NCI’s approach to evaluating plant-derived anticancer compounds. In addition, the ISO 10993-5 standard for in-vitro cytotoxicity states that a material is considered non-cytotoxic when cell viability remains at or above 70% of the untreated control, with lower values pointing to cytotoxic activity^46^.

In the present study, the cell cycle distribution was studied on A459 cells treated with neem extract at its IC_50_. The results indicated that A549 treated with doxorubicin induced arrest of cell cycle at the G1 and G0 with cell population percentage of 62.37% and 34.21%, respectively. While, neem extract induced arrest of cell cycle at the G0 phase only with cell population percentage of 96.89%.

Parallel results were obtained by Santos et al. (2023) and Madhayan et al. (2021)^47,48^ who showed significant accumulation of cells in the G0/G1phase of the cell cycle. All together lead one to suggest that neem extract can induce apoptosis. Also, the findings of study of Hao et al.^49^ suggest that treating of HeLa cells with neem extract decreased the concentrations of cyclin B, cyclin D1, and increase the CKI, p21 expression, which cause the arrest of cell cycle at G0/G1, which may be the case in the present study.

Bax is the first known member of proapoptotic Bcl-2 genes family. It has a vital role in enhancing apoptosis^50^. In the present study, BAX, Bcl-2 and P53 as a proapoptotic, anti-apoptotic and tumor suppressor genes were evaluated.

The results proved that, the treatment of A549 cells with neem leaves extract downregulated the level of Bcl-2 gene and upregulated BAX and P53 gene. From these results, we can suggest that neem extract mediated the apoptosis process which may be due to upregulation of immune surveillance and thence increment in macrophage activity.

The results of Tiple et al.^43^ were similar with us as they reported an enhance in the expression of Bax in neem treated MCF-7 and HeLa cells in a time-dependent manner but lower gene expression was observed in the untreated cells. Another study which was constructed by Santos et al.^44^ which evaluate the apoptotic proteins levels after administration of neem extract in a dose of 200 mg/g three times per week. They showed that, administration of neem extract was found to increase Bcl-2 protein and increase both of caspase 8 and caspase 3 proteins levels in an animal model. The apoptotic inducing effect of neem leaves extract was found to improve both humoral and cellular immune responses^44^.

Additionally, the study of Madhayan et al.^48^ support our results in which neem extract treatment inhibited the level the antiapoptotic bcl-2 protein and also enhance the level of Bax protein. Also, the results of Schumacher et al.^51^ proved that neem extract gradually induced apoptosis as the concentrations of the anti-apoptotic proteins Bcl-xL, Bid, and XIAP were decreased after the neem extract treatment. Further, in the study of Kumar et al.^52^ the PC-3 cells treated with ethanolic neem extract showed decline in the concentration of Bcl-2 protein and increasing in BAX protein level in a dose-dependent manner.

## Conclusion

From the results of this study, it was found that neem leaves extract was able to inhibit cancer cell lines proliferation via inducing arrest of cell cycle at G0, stimulate apoptotic genes expression and decline the anti-apoptotic genes expression. It also has a strong antimicrobial and anti-oxidant activity. Its high polyphenolic content plays a role of the presented activities.

## Data Availability

The data generated during and/or analyzed during the current study are available from the corresponding author on reasonable request.
